# EMDR in pediatric hospital setting: a case report of an adolescent with cancer

**DOI:** 10.3389/fpsyg.2024.1347822

**Published:** 2024-02-22

**Authors:** Sabrina Ciappina, Elvia Roccia, Deborah Concas, Elisa Faretta, Isabel Fernandez, Paola Quarello, Giulia Zucchetti, Franca Fagioli

**Affiliations:** ^1^Pediatric Oncology Department, Regina Margherita Children's Hospital, AOU Città della Salute e della Scienza, Turin, Italy; ^2^EMDR Italy Association, Bovisio Masciago, Italy; ^3^Department of Sciences of Public Health and Pediatrics, University of Turin, Turin, Italy

**Keywords:** pediatric oncology, cancer, EMDR, psychotherapy, pediatric hospital, case report, adolescents

## Abstract

**Introduction:**

Childhood cancer is rare, but it is the most frequent serious event with a high risk of traumatization for children, adolescents, parents and siblings. EMDR is widely studied as clinical intervention that addressed cancer-related stressors among cancer adult population, but to our knowledge, no researches have been conducted among children and adolescent with cancer.

**Methods:**

The purpose of this case study is to describe for the first time the application of the EMDR protocol in a pediatric hospital setting with a 17-years-old Italian adolescent who received a diagnosis of leukemia. He accessed the psychological support service complaining of feelings of anxiety and general discomfort. EMDR protocol started after the diagnosis and ended after the usual eight phases. The Impact of Event Scale—Revised (IES-R) was used to assess stress disorders symptoms as outcome at the baseline (before the First Phase) and at the end of the EMDR protocol (after the Eight Phase).

**Results:**

By using EMDR protocol the patient reported a decrease of emotional activation after a few EMDR sessions.

**Conclusion:**

EMDR protocol may be effective for pediatric cancer patients in treating stress disorders symptoms and it can be proposed immediately after diagnosis as a standard care also in pediatric hospital setting.

## Introduction

Cancer in children or adolescents is rare, but it remains the most frequent serious event in the pediatric age group. Every year, about 2,300 new cases of childhood cancer are recorded in Italy: 1,400 cases in the 0-14-year age group and 900 cases in the 15–19-year age group (AIRTUM Working Group, 2013). In the pediatric oncology field, oncological diagnosis represents a traumatic event not only for patients but for the entire families (Faretta et al., 2016; Van Warmerdam et al., 2019; Zucchetti et al., 2022). It evokes a sense of threat to a person’s sense of vulnerability, or a loss of control, a sense of helplessness (Mercadante et al., 2004; Carlsson et al., 2019). Growing body of research indicates that posttraumatic stress disorder (PTSD) and symptoms (PTSS) are some of the most important psychological consequences for those affected by childhood cancer (Portigliatti Pomeri et al., 2021), as well as their parents and siblings (Tremolada et al., 2016; Koutná et al., 2022). The traumatic experience of cancer illness usually starts at diagnosis communication, continuing throughout the course of care until the end of treatment and beginning of the survivorship phase (Zucchetti et al., 2023). Every moment of the oncological journey represents a potentially traumatic experience: the first access to the hospital/emergency room, receiving test results, diagnosis communication, the beginning of treatment, hospitalization, physical changes, surgical intervention, disease relapse and, for some, also the palliation phase. Common symptoms during these moments are post-traumatic stress, anxiety, mood and sleep problems, flashback, frequently worries, hyper-arousal, negative cognition and mood, anxiety, depression and fear (Lewandowski et al., 2011; Pinquart and Shen, 2011; Marusak et al., 2019). During adolescence these symptoms can get worse since that adolescence is the most delicate phase of life for all the development tasks and cancer diagnosis in this stage of life can interrupted or undermine the growth’s process. Adolescent with cancer must interrupt school attendance, have to stay isolated because of illness, have to give up seeing friends, have to face physical changes. The most delicate thing they face, however, is the fear of illness and the fear of death. Fear of cancer recurrence or progression, anxiety and depressive symptoms are frequently reported problems among adolescent’s cancer patients (Sun et al., 2019). Nonetheless, particularly during the survival phase, have also been described positive consequences named as posttraumatic growth (PTG) and occur in the domains of personal strength, relating to others, appreciation of life, new possibilities and spirituality (Turner et al., 2018). Psychosocial support is provided to adolescents with cancer by psychologists who follow the main guidelines of the psychoncology field implemented in the main center of the Italian Pediatric Hematology Oncology Association (AIEOP). This support, that consists in individual psychotherapy, psychoeducational intervention and/or clinical group support is provided routinely from the moment of the diagnosis, during treatment and after the end of the medical treatment (Zucchetti et al., 2023). Also, to support this delicate stage of life, many educational and recreational activities are offered to adolescents such as sports. From literature we known that the benefits and the skills learned from these activities, in addition to having a positive effect on social connection (Sebri et al., 2019; Durosini et al., 2021), can be transferred to other contexts that concern the psychological sphere such as body image and romantic relationships by also reducing typical negative symptoms such as for example stress experienced by cancer adolescents. In addition to these successful interventions for adolescents cancer patients, some studies are highlighting the potential of an innovative approach that is the Eye Movement Desensitization and Reprocessing (EMDR). This type of intervention has significant effect on stress symptoms among other populations, such as oncological adult patients (Capezzani et al., 2013; Faretta, 2014, 2018; Faretta and Civilotti, 2016; Faretta et al., 2016). To date, to the best of our knowledge, no studies have investigated the efficacy of EMDR therapy in the treatment of stress disorders symptoms experienced from cancer adolescent. The purpose of this case study is to describe for the first time the implementation of EMDR protocol for the treatment of a male cancer adolescent patient admitted for cancer treatment in a pediatric hospital setting. Our main hypothesis is that EMDR can be effective in reducing stress symptoms. CARE guidelines for writing a patient case report in a checklist were used to enhance the manuscript process (Supplementary material 1).

## Patients information

Enrico (pseudonym) was diagnosed with a rare form of childhood leukemia and he was admitted at the Pediatric Oncology Department of the Regina Margherita Children’s Hospital, one of the main pediatric hospital in Italy (Zucchetti et al., 2018). Socio-demographic characteristics of Enrico are: 17-years-old adolescent, he is a student, very dedicated and interested in school. He is an only child, he lives with his caregiver. Socio-demographic characteristic of his caregiver are: Enrico’s mother is 45 years old, she is an educator. Enrico’s father is 48 years old, he is an employee. They are married, both high school graduated. Place of residence is out of city. No significant event reported in last years. Enrico’s medical treatment includes lengthy hospitalization for chemotherapy treatments and pain therapy. The course of treatment was expected to last about 2 years. Enrico and his family are offered the participation to the clinical and research psychological protocol named EMDR_ITA_PED approved by the Ethics Committee of AOU Città della Salute e della Scienza of Turin (Prot. No. 0073656; July 2022). The protocol provides in addition to standard psychological support also the EMDR treatment. Inclusion criteria are: patient diagnosed with oncohematology disease; age range ≥ 12 years old; acceptance informed consent. Exclusion criteria are: patients <12 years old; no acceptance of informed consent. All procedures performed were run following ethical standards of the institutional and/or national research committee. Before the study we informed patients and families about the aim and procedure of protocol EMDR Therapy and obtained their informed consent. For the Enrico’s treatment we followed the EMDR Protocol proposed by Faretta et al. (2016), a specific protocol for cancer focused on difficulties related to different stage of the illness (Shapiro, 2001; Murray, 2010). The EMDR procedure was explained and we obtained consent for the treatment. No pharmacological treatment and other type of psychotherapy were provided before. The following is a brief description of the steps that will be followed:

*Phase 1: Client history -* follows the standard EMDR protocol, with an increased focus on the self-disease relationship and significance of the disease in the patient’s history.

*Phase 2: Preparation -* follows the standard EMDR protocol, including time dedicated to psychoeducation on pain and oncological illness.

*Phase 3: Assessment -* this is the only phase different from the standard EMDR protocol. Targets are related to traumatic experience due to illness, and to concerns and current issues (surgical intervention, treatments, hospitalization…).

*Phase 4: Desensitization and reprocessing -* follows the standard EMDR protocol. In this phase the role of therapist as a “safe base” for patients is very important.

*Phase 5: Installation -* follows the standard EMDR protocol and integrates the installation of positive cognition.

*Phase 6: Body Scan -* is identical to standard EMDR procedure.

*Phase 7: Closing the session -* includes the imagery of health resources.

*Phase 8: Re-evaluation* - follows the standard EMDR protocol.

## Diagnostic assessment

### Quantitative measures: self-report questionnaire + time points

Psychological assessment was agreed with the EMDR Italy Association and then approved by the Ethics Committee of the AOU Città della Salute e della Scienza of Turin (EMDR_ITA_PED, Prot N. 0073656). The protocol provides an examination and assessment of each patient before the treatment in order to evaluate the appropriateness of the treatment and after the treatment in order to verify the effectiveness. Selected outcomes were collected at the beginning of the EMDR protocol (Time 0), and at the end of the protocol (Time 1), through the administration of some test. The first is the Impact Event Scale-Revised (IES-R), that is a 22-item self-report scale that measures subjective exposure to traumatic experiences with satisfactory values of internal consistency (overall Cronbach’s alpha for the total IES-R was 0.94). The questionnaire requires to participant the naming of a specific stressful life event and then to indicate how distressed they have been by each listed stressful life event in the past 7 days on a 5-point scale. The other test we propose at Time 0 is the Distress Thermometer, a screening tools for measuring distress on a range 0–10 point Likert Scale. This scale includes also 4 sub-scale screening following problems: practical, family, emotional, physical, and spiritual. At Time 1 we proposed the same screening test as in Time 0 with the addition of the Post-Traumatic Growth Inventory Test composed by 21 items to assess positive outcomes reported by subjects who have experienced traumatic events using a scale ranging from 0 to 5. A higher score indicates a higher level of posttraumatic growth. Results about Enrico’s assessment at Time 0 are: 8 at the Distress Test and more problems on a subscale of Emotive and Physical problems. At IES-R test results shows high scores on a subscale of Intrusion (= 3 on a range 0–4) and moderate symptoms in other subscale (Avoidance = 2,6 Hyperarousal = 2,75 on a range 0–4).

### Therapeutic intervention

**Figure 1:**
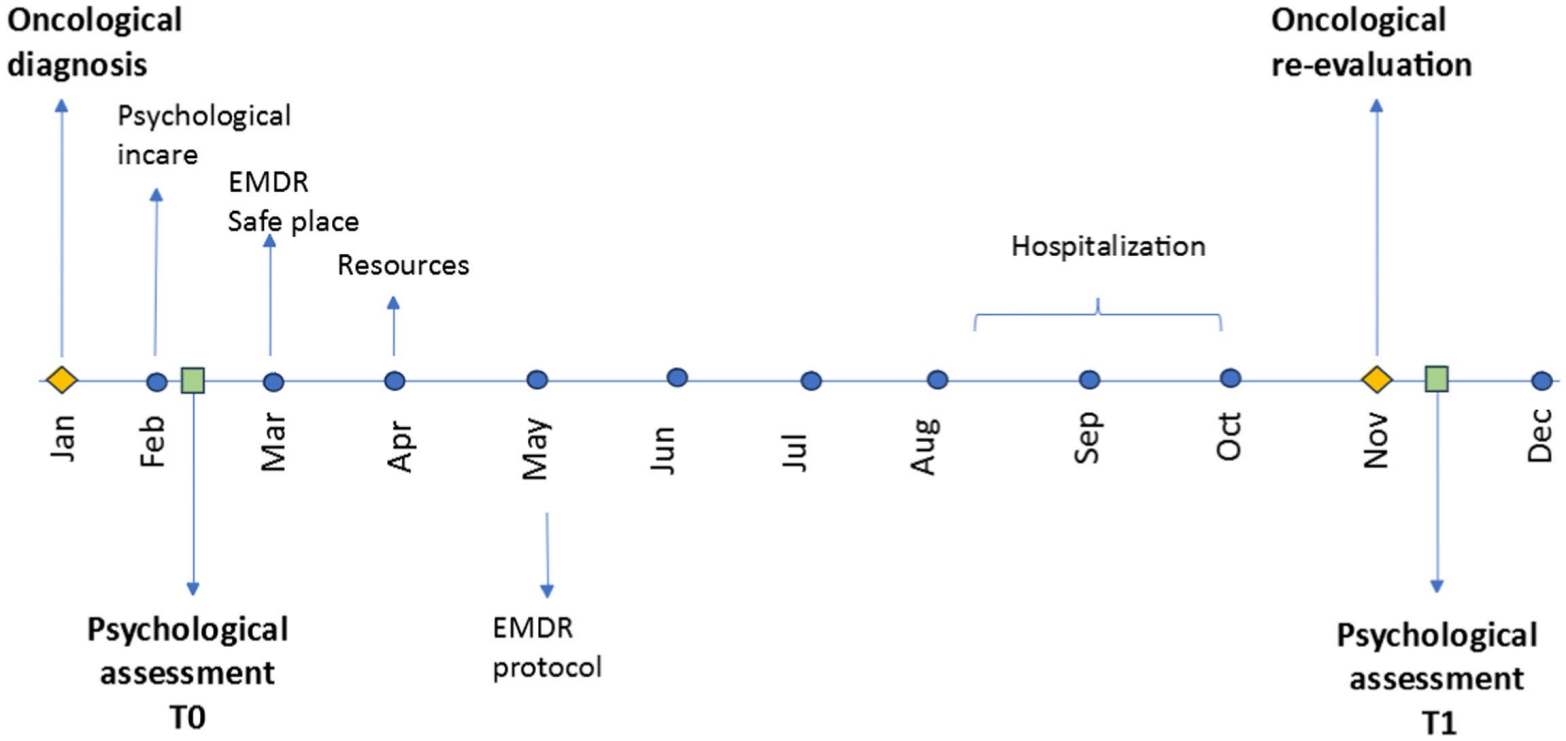
Time points.

The EMDR protocol stared with Enrico immediately after the diagnosis communication. The intervention was proposed by the psychologist of the Pediatric Oncology Department, trained in the use of the EMDR method and supervised by the EMDR Italy Association. We proposed EMDR therapy also to his caregiver. Enrico’s mum started EMDR therapy at oncological diagnosis but her psychotherapeutic course was not continuous. She continued to work and only on a few occasions accompanied E. to inpatient care. His father never expressed the need for psychotherapeutic support, but we only worked with him at a first level of intervention. Table 1 explains the specific content of EMDR intervention’s phases and Figure 1 shows the stages and process of EMDR protocol by highlighting also some important time points of Enrico’s course of treatment.

**Table 1:** Description of EMDR intervention phases.

**Phases**	**Specific content, goal and reference**
First phase: building therapeutic alliance and psychoeducation on the EMDR method	Psychologist during the first phase focused on building a positive relationship with patients. Explain EMDR methods doing psychoeducation and focuses the first few moments on stabilizing and focusing resources. Our goal in the early stages is to provide concrete help and try to stabilize patients, reinforce coping skills to restore a sense of self-efficacy. We collect the patient’s personal history and begin to prepare our work on the traumatic events related to the disease. No significant events are reported. We proceed with the installation of safe place and RDI. E. reported about the image of a mountain immerged in the nature as a safe place. He would like to feel able to cope with the situation so he reported three episodes in which he felt capable.
Second phase: assessment	This phase is proposed in line with the specific protocol for cancer patients (Faretta, 2014), so target are related with cancer illness. We also administer test (IES-R and Distress Thermometer) as per protocol (described in methodology section) to have a more comprehensive assessment than the current emotional state of patients. To help Enrico the psychologist invited him to talk about his fears and most frightening thoughts related to what was happening (cancer illness). We invite patients to start with the most traumatic event. E. requires help about his anxiety due to the illness.
Third phase: target selection	Targets are related to traumatic experience due to illness, and to concerns and current issues. During this research he remembers from the first access to the hospital. Negative cognition from which we start is “I am helples*s,”* Positive cognition is *“I can do it.”* SUD = 8 VOC = 3.
Fourth phase: desensitization and changement	We start with desensitization with BLS. At the end SUD was 1 and VOC was 7-. He felt stronger and more appreciative of his own skills.
Fifth phase: installation of Positive Cognition (PC)	In this phase psychologist installed PC and asks the patient for feedback. E. felt himself stronger.
Sixth phase: body scan	Psychologist check whether the patient still perceives any negative feelings or distress. In body scan phase we did not meet any difficulties or barriers.
Seventh phase: closing and check	In this phase psychologist check patient’s psychological state. We closed surely that E. was in a safe emotional condition.
Eighth phase: re evaluation	We conclude our treatment monitoring other moments of the course of care in order to continue an instantaneous processing of the potentially traumatic event. We conclude with the administration of test (IES-R, Distress Thermometer and Post-Traumatic Growth Inventory).

#### First phase: clinical assessment

Initial meetings with E. were focused on creating a therapeutic alliance. He was open to talking about his feelings of anger, mood changes and frustration. At an early point, E. reported specifically worries about schooling for the future months. In Italy, patients undergoing treatment cannot attend school due to the risk from the immunosuppression effect of oncology treatment. However, we proposed that E. participate in our Hospital School during treatments. Enrico cared a lot about school and while very organized he felt extreme fatigue during cancer treatment. He was afraid he would be unable to keep up with his studies, leading to significant anxiety. In order to not fall behind, he was forced to give up time usually devoted to his hobby and passion for music, and this created strong frustration in him. He shared that he often felt angry and volatile and no longer recognized himself. Enrico feared losing control. His caregivers appeared to be having some difficulties managing this situation, especially his mother.

#### Second phase: preparation

Identifying a patients’ needs is part of phase one, through a good therapeutic alliance, and to reinforce emotional coping skills to restore to the patient a sense of self-efficacy and hope, safety and network support. E. needed help with his anxiety and feelings of not being able to do everything. His principal need was to recover his sense of efficacy to feel able to fight the situation. We used a psychoeducation intervention to explain the connection between his feelings and the oncological illness to help strengthen his sense of control of the events. We proceeded with the installation of a safe place for these purposes. E. chose the image of an isolated mountain lodge immersed in the green of nature connected to the word and feelings of well-being. With the aim of strengthening the self-help ability of E., we proceeded with resource installation. E. was feeling severely distressed by the disease and related physical changes leading to him feeling weak, incapable, tired, and defenseless. During Resource Development and Installation (RDI), E. identified three episodes when he felt confident after playing a concert. He reported that after overcoming a challenge during the concert he felt happy and relaxed. The first episode he referred to was a concert in which he performed with 3 other people. He was very anxious but it was a great experience; he was satisfied with his performance, he felt capable. He connected this memory with the words I’m capable. The second episode was a final exam at school, “the first big hurdle to be faced” for him: He described working hard despite his fear; his strength helped him and he was able to persevere and achieve success. He connected this memory with the words I can do it. The third episode of confidence was a relational resource: the relationship with his best friend. E. felt relaxed, safe, happy and carefree. With his friend he was able to stop worrying and he felt free. We used RDI to install a memory of a chess match when they laughed and he felt serene. E. connected these memories with the word joy.

#### Third phase: target selection

In this phase, dedicated to target selection, E. had to define clusters related to trauma connected with the experience of cancer illness. We asked him to identify the event and formulate a negative belief about himself (NC). The choice of target was a delicate moment for E. because in this phase he was suffering due to his treatment and medicine; he suffered from anxiety and, especially at night, he had severe crying fits, breathing difficulties, and moments of strong anger that his mother tried to help him contain. In the morning he was quieter so we could work on his traumatic memories. During the search for triggers, he remembered the initial moments of his arrival at the hospital, starting with communication of the diagnosis when the doctor told him what his treatment would be. The traumatic imagines he evocated: the *oncologist at the door ready to go out while he remained still in the bed,* and *himself after the doctor went out, crying and banging his fist against the pillow.* He chose the first worst images to be connected with the negative cognition *I am helpless.* His emotions were anger, frustration, and a strong weight on the chest. Positive cognition was *I can do it.* Perceived validity (VOC) = 3, Subjective Units of Disturbance Scale (SUD) = 8.

#### Fourth phase: desensitization and reprocessing

In this phase, we invited E. to undergo desensitization through Bilateral Stimulation (BLS). This is a delicate phase for patients, where a trusting relationship and therapeutic alliance are very important. E. used two sessions for this phase. Each session took 6 sets (using the wireless kit). During the first session, he reported a more vivid memory from which the mind tried to detach itself and return to its worst image, with the mind trying to escape (*Now the memory is more vivid; I’m a little more troubled, weighed down; my mind was trying to detach, random images...happy moments, the image is heavy; When I thought about anger, the image of me banging my fists against the pillow came back; Nothing new; My mind tried to escape*). At the end of the session, he reported SUD 8 and VOC 4. At the beginning of the second session, we verified that the SUD was 7 and VOC 5. After BLS, E. struggled to visualize the image and also the memory, which appears more blurred. The initial anger had turned to sadness. He was still struggling to relax (*Nothing, it seems less strong, I visualize it less; I struggle to visualize the memory, everything; I feel bitterness, sadness, it brings me back to the fact that I’m here, it’s nothing new, I do not feel anger; I cannot focus on the memory, I’m not really relaxed right now*). At the end, the recorded SUD was 3 and VOC was 5. He said: *I realize actually, not...about the situation, but about the treatment, having to be here; My mind is not at all vulnerable today.* At the end of the last BLS, SUD was 1 and VOC was −7. *The minus figure is because in fact I have to stay here and that makes me think a little bit.*

#### Fifth phase: installation of positive cognition

Having reached SUD 1 and VOC 7-, accepting that minus as important but not a limitation to processing, we installed the positive cognition chosen and confirmed *I can do it* despite everything. The feedback during the installation was positive. E. felt stronger and more appreciative of his own skills.

#### Sixth phase: body scan

In recalling the event and focusing on PC, we focused on his body sensations to check for unpleasant sensations indicating distress. In this phase, we did not meet any obstacles and therefore proceeded with the BLS. E. was completely free of somatic tension or unpleasant feelings, instead feeling relaxed.

#### Seventh phase: closing the session

At this stage, when the session was complete and having ascertained that E. was in a safe emotional condition, we proceeded with a relaxation technique achieved through breathing and returning to the safe place.

#### Eighth phase: re-evaluation

In this final phase, E. did not make any reference to dreams or meaningful thoughts. In this first phase of cancer treatment, we focused our attention on the diagnosis communication, but significant moments of the illness were also monitored in order to continue an instantaneous processing of the traumatic event.

## Outcomes

Table 2 shows the results of the EMDR Protocol between the two waves and the reduction of the stress symptoms in the patient at Time 0 and at Time 1. Results highlight at Time 0 high mean scores and close to the value for the presence of PTSD symptoms (Mean ≥ 3 on a range 0–4). Subscale that showed more important emotional activation at Time 0 is the Subscale of Intrusion (Mean = 3); Between Time 0 and Time 1 there is a reduction in emotional impact in all Subscales but especially in the Subscale of Intrusion (Δ1,4). Distress Thermometer shows high scores at Time 0 (=8) that decrease significantly at Time 1 (=4). On a Scale of Problem List, emotive problems are those most frequently reported at Time 0 (=6/6) and decreasing at Time 1 by half (=3/6). Furthermore, at Time 1 through the administration of the Post-Traumatic Growth Inventory, E. refers to perceive himself stronger and to have cultivated new interests (= 5).

**Table 2:** Clinical findings across time points.

	**T0**	**T1**	Δ
IES-R *Subscale Intrusion*	3	1.6	1.4
*Subscale Avoidance*	2.6	2	0.6
*Subscale Hyperarousa*l	2.75	1.8	0.95
Distress thermometer Problem list	8	4	4
Emotive items	6 YES/0 NO (6/6)	3 YES/ 3 NO (3/6)	3
Practical items	2 YES/ 5 NO (2/7)	2 YES/ 5 NO (2/7)	0
Family items	1 YES/3 NO (1/4)	0 YES/4 NO (0/4)	1
Spiritual items	0 YES/ 1 NO (0/1)	0 YES/ 1 NO (0/1)	0
Physical items	7 YES/ 15 NO (7/22)	6 YES/16 NO (6/22)	1
Post traumatic growth inventory	–	5 (on a range 0–5) about: *feeling strong, cultivate new interests*	–

## Discussion

Oncological diseases, especially if experienced in pediatric age, expose young children and adolescents to conditions of serious physical and emotional stress causing delays in their physical development, cognitive acquisitioning and issues related to their emotional and social functioning. It also represents a traumatic experience for the entire family and a disruption in daily family life. Pediatric oncological illness is a high-risk traumatization and retraumatization event that affects patients in their global identity: it generates a threat to the quality of life and psycho- physical integrity of the patients and family members, strong emotional reactions emerge with high arousal, avoidance, and intrusive thoughts. Recent studies underlined that especially adolescents with cancer experience anger feelings and dissociation. For all these reasons, the risk is the development of PTSD symptoms, not necessarily related with the histology or duration of treatment. Since that research highlighted that cancer or cancer- related events are experienced by children and adolescents as traumatic and stressful event proposing dedicated interventions, it is not merely desirable but actually essential in psychooncology. As demonstrate in other important studies (Elmagd et al., 2018; Sebri et al., 2019; Durosini et al., 2021) reducing psychological disfunction through innovative supportive intervention can be the first step to increasing psycho-physical well-being, especially during cancer treatment, as long as the possible medical limitations are taken into consideration. EMDR therapy can be an effective and innovative strategy to fight stress feelings and sensations that children and adolescents experience during their oncology journey. For these reasons, we have seen that the faster we act in processing, starting with the communication of the diagnosis, the more resources patients will have to succeed in dealing with the later stages of the disease journey. EMDR therapy in fact seems to help patient, also during the adolescence stage, to deal with a stressful experience such as cancer. In addition, our data are in line with the most recent literature on the application of EMDR in psycho-oncology in the reduction of intrusive symptoms and also, for a global well being in a long run. As the literature suggests early interventions to prevent and process traumatic emotional experiences can prevent the onset of long-term posttraumatic stress disorder (Capezzani et al., 2013; Faretta et al., 2016). Our research has not encountered any major limitations so far, with the only possible issues relating to conditions of patients (fever, nausea, fatigue, contact isolation), in particular during hospitalization and the hospital setting because often patients have to stay in their room during hospitalization due to chemotherapy treatment, and healthcare workers often enter this room, thus interrupting the session. Also, since there are no controls, we could not assume that the EMDR protocol is more effective than usual care. Despite these limitations, EMDR could be considered as a potentially useful adjunctive treatment for adolescents who suffering from traumatic experience such as cancer disease. In the future the goal will be to expand EMDR use in the pediatric hospital setting for pediatric patients with EMDR adolescent’s group for example, and also with other specific and traumatic situations such as palliative care or with Ukrainian oncological pediatric patients as a daily standard practice.

### Patient perspective

Enrico at the end of the intervention trusted the method, reporting a decrease in intrusive thoughts and a reduction in fearful emotions thank to EMDR Protocol. He said he is more aware of his own way of experiencing the emotions related to that traumatic event.

## Data availability statement

The raw data supporting the conclusions of this article will be made available by the authors, without undue reservation.

## Ethics statement

The studies involving humans were approved by Ethics Committe AOU city of health and science of Turin. The studies were conducted in accordance with the local legislation and institutional requirements. Written informed consent for participation in this study was provided by the participants’ legal guardians/next of kin. Written informed consent was obtained from the individual(s) for the publication of any potentially identifiable images or data included in this article.

## Author contributions

SC: Writing – original draft. ER: Writing – original draft. DC: Writing – original draft. EF: Writing – original draft. IF: Writing – original draft. PQ: Writing – original draft. GZ: Writing – review & editing. FF: Writing – review & editing.
